# Yellow Fever

**Published:** 1873-04

**Authors:** T. A. Cook

**Affiliations:** Louisiana


### SOUTHERN MEDICAL RECORD.

VOL. III.—APRIL, 1873.—NO. 4.

### PART I.

### ORIGINAL COMMUNICATIONS.

### YELLOW FEVER.

BY T. A. COOK, M.D., OF LOUISIANA.

A statement of facts connected with the yellow fever epidemic which occurred in the town of Washington, and at Port Barrè, in the parish of St. Landry, Louisiana, in the fall of 1870, is due to the medical profession. This duty I will endeavor to discharge as succinctly as the subject will admit of.

In the year 1859, some three hundred free colored people emigrated from this parish to the island of Saint Domingo. Most of them were in independent circumstances—none were in want. They were creoles of this country—born and reared in this parish—in almost every instance presenting a mixture of race, generally of the white and black, and in some cases showing the distinctive marks of the Mexican, Spaniard and Indian. Not long after their arrival on the island, a large number of these people died of yellow fever, or of what was called by the residents, *La Fievre Hemorrhagique*. They remained in the island until the latter part of July, 1870, having experienced the saddest vicissitudes of fortune, and having interred fully two-thirds of their original number. At this time a United States vessel was ordered to transport them back to Louisiana. On the arrival of the vessel at the port of New Orleans towards the end of July, they, with their baggage, were immediately transferred from the public vessel to a steamer, which was on the point of leaving for Washington. They remained in New Orleans only so long as was required for their transfer on board the steamer, and this, it is said, was accomplished in about two hours. Their baggage was stored away in boxes and trunks. At Barrè—a port of the parish, on the same stream with Washington, some ten miles east—about twenty-five of the returned emigrants debarked, and stored their baggage in one of the warehouses of the place. The remainder—seventy in number, more or less—came on to Washington, and domiciliated themselves in nearly the centre of the town, on a lot on which were several unoccupied houses. About the 10th of August, they unpacked and hauled their baggage (which now was the source of a nauseating effluvia) near a quarter of a mile to the bayou Carron—the southern boundary of the town—for the purpose of a general washing. In their progress they sowed the pestilence itself, for, in less than two days, all the unacclimated in the immediate neighborhood of the lot on which they had settled, as well as on the street leading to the bayou, were stricken down with the fever—especially inmates of dwellings near the place where the baggage was exposed to the air. In seven days from this time, there died in the localities mentioned six persons, all having unmistakable symptoms of yellow fever. It was fortunate that three-fourths of the people thus directly exposed to a concentrated infection had, in previous epidemics, had this disease. It soon spread over the town, sparing none but those who had previously had it. Notwithstanding this violent outbreak of the epidemic, no one had suspected the danger we were subjected to from the emigration from Saint Domingo, for the fact that these people were in the enjoyment of health—having all had the yellow fever in the island—and the withdrawal from the public eye of a large amount of boxed-up baggage, disarmed all suspicion of the impending peril.

Port Barrè is a small settlement, but, being a point of considerable export and import for a large extent of country, a great many people in the business season visit the place. At the time of the arrival of the emigrants, luckily, the business season was over; but curiosity, and a desire to hire laborers, induced the neighbors to visit the emigrants. No epidemic of any kind had, up to this time, visited the place. But, simultaneously with the outbreak in Washington, this fever, in a fatal form, appeared at, and in the immediate neighborhood of, Barrè. In nearly all the fatal cases black vomit was present. The disease, it appears, was limited to those who may be said to reside in the place and to visitors. The town of Washington, subjected to no recognized cause of disease, has ever been remarkable for its healthiness, with the exceptions of the epidemics of yellow fever which have visited the place four times. It is situated on the south bank of the Courtableau, a constantly-running stream, on an undulating ridge, and is not exposed to stagnant waters from any quarter.

Having now called the attention of the readers of your journal to this isolated epidemic of yellow fever on the continent of North America, and to the cause the only antecedent to its occurrence—the arrival of the returned emigrants from Saint Domingo, with baggage which appeared to have been saturated with the poison of yellow fever—I might leave to the profession to draw the unavoidable inferences which the facts indicate. But, being conversant with the circumstances which attended the breaking out of this disease in its previous visitations to this region of Louisiana, I shall, I hope, be pardoned for giving my experience more extensively, of telling what I have seen along with our whole people, and of making such comments as facts may elicit.

Before 1837, cases of yellow fever had been seen in this parish, and, most probably, in other parishes having water communication with the city. Flat-boat loads of goods had been occasionally shipped from New Orleans during the prevalence of yellow fever, and, on being opened, had communicated the disease—and this was well known to have been the case on two occasions in Opelousas. It is recorded that about 1828 and 1832, persons exposed to goods opened in the town, and direct from New Orleans, died with black vomit. But, notwithstanding the importation of the *poison in goods*, no epidemic followed. It was reserved for the year 1837 to unfurl for the first time the yellow banner of this atrocious disease—for the people of Opelousas to experience for the first time the desolations of yellow fever. This epidemic followed the importation of some goods and baggage, and of their owner, who fell sick immediately on his arrival in the town. It was a new disease to the physicians and to the people, and, from its resemblance to the hot fevers of the country, both the people and physicians, at first, fell into errors as to the treatment—and the fatality was appalling.

Also, in 1839, the introduction into Opelousas of both infected baggage and merchandise, and of a yellow fever patient, preceded the breaking out of the epidemic, which this year did not spare a single unacclimated resident of the town. The mortality was the very maximum ever witnessed during the general prevalence of any disease. But so prevalent was the fever that no extensive epidemic could possibly have been engendered in this town for some years subsequent to 1839. In 1842, in the latter part of October, a merchant residing in the then outskirt of the town, imported infected goods from the city. In the first week of November, his young clerk, not long before arrived from France, was taken down with a fatal attack of fever. So difficult is it to distinguish the yellow from the common fevers of the country, that I was opposed by my *confreres* in my diagnosis of this affection, until black vomit branded it as a disease not to be mistaken. Some twenty-five cases followed, when, as usual, the occurrence of frost completely extinguished the disease. Since 1842, a quarantine, the most rigorously enforced, has been established for the protection of this town so soon as any apprehension was felt of the danger of importation, and though during this long time Vermilionville and Washington, contiguous towns, have been theatres of fatal epidemics, Opelousas has been quite exempt from the disease.

As to the epidemics in Washington in the years 1853, 1854, 1867 and 1870, no one conversant with the facts preceding them doubts the importation of the cause. In two of these epidemics, the owners accompanying the infected goods and baggage were the first—in fact, were sick on their arrival in town. In 1853 and 1870, the parties who came with their goods and baggage had previously suffered with this fever. In these two instances, fatal cases immediately followed the ventilation of the baggage. It is hardly conceivable that this connection between the importation of goods previously exposed to an infected atmosphere, and the subsequent epidemic, was merely accidental—a mere concomitance of events. In 1870, far away in the interior of Western Louisiana, before public attention had been called to the disease anywhere else on the continent, two small villages, having received within their limits some two weeks previously emigrants from Saint Domingo, are severely stricken with an epidemic of yellow fever. It appears to me there could not be a clearer demonstration of the importability of the cause of yellow fever, than was manifested in the origin of the two epidemics in the localities specified. Each place, under the same climatic influences, enjoying a reputation in general for salubrity, was visited at the same time by a disease marked by identical symptoms, running the same course, and presenting all the phenomena of yellow fever. These two places alone were the receptacles of emigrants, with all their household chattels, from Saint Domingo. In these two places alone the epidemic raged.

In 1870 a gentleman, in the midst of the epidemic in Washington, hauled to his store, about ten miles distant, a wagon-load of goods embracing a lot of coffee. There were present at the opening of most of the goods, besides the proprietor, his son, clerk and a boy living on the premises, four persons from the immediate neighborhood. These eight individuals all fell sick with the same fever almost in the same hour; and three others who visited the store a day or two after, also in a few days were stricken down. I do not think I ever beheld yellow fever assume a more repulsive aspect than it did in these cases. The mortality, as is generally the case in the country, was extreme—fifty per cent. I have been told that the occurrence of the cases in the little village of Ville Platte, the same year, followed the immediate importation of goods from Washington. It may be here stated that this disease, as a rule, never occurs primarily in the interior towns, but subsequently to its prevalence in some commercial emporium. The epidemic in 1870 hardly constitutes an exception to the general rule. Another most noticable fact: In 1853, in the month of August, the remnant of a large mulatto family, that had, about twelve months previously, left this town and settled in New Orleans—survivors of the ruthless disease which scourged the city that year—returned to Washington with trunks and boxes of baggage. These trunks, etc., were deposited in the store of Simon Lepp. About ten days elapsed before any one would touch them. They were finally opened, and the odor emitted was nauseating in the extreme. Besides two unacclimated clerks, there were accidentally present on the occasion three respectable individuals from the country. The clothing was hung over the picket fence separating Lepp's premises from those of P. Sterling. Mrs. Sterling and a member of her family were subjected, by direct contact with this clothing, to the effluvia emanating from it. These seven individuals very speedily and about the same time fell sick—to wit, the three persons from the country, the two clerks of Lepp, and the two members of Sterling's family—and five of them died with black vomit in less than four days from the commencement of their attacks. The breaking out of the fever in 1870 was precisely similar, exhibiting clearly the fact that these closely boxed-up goods could be conveyed from place to place with safety, and that they imparted no disease until the goods and baggage were exposed to air, when would be manifested the presence of a deadly poison, affecting fatally, and almost instantly, all liable persons exposed to it. The idea that the importation of cases must precede its diffusion, is altogether unfounded. It is admitted by writers on the subject of transmissible diseases—that is, of their causes—and especially of the contagions, that the poison is conveyed from one point to another through merchandise or baggage. The spontaneous development of small pox, for example, is, I believe, generally denied by the profession. The doctrine is held of an unbroken propagation of this affection, sometimes through diseased persons, but most generally through the medium of fomites.

That the cause of yellow fever is transmissible, we have cited facts, which we have seen with our eyes, that must be received as satisfactory proof. Hundreds of other instances might be cited in corroboration. Nor does it appear to me that the doctrine of its importation is at all weakened by a failure in city or towns to point out the specific importation. It must, necessarily, often happen that this detection is difficult, if not impossible. We have seen, in 1870, a cargo of yellow fever brought into the port of New Orleans, and, before its diffusion was possible, transferred to a steamer and conveyed in part to Port Barrè, and in part to Washington, in St. Landry parish, Louisiana, where, being landed, there ensued a fatal epidemic in both places. The importation and the speedy breaking out of the disease must be regarded in the relation of cause and effect. Even after the establishment of summer heat, vessels often arrive from places where, notwithstanding the prevalence of the cause, no epidemic, not even cases of yellow fever exist, for the reason that there are in such places no unacclimated persons.

If the facts be as stated, the question naturally arises, Why do New Orleans and the interior towns enjoy for a succession of years an exemption from this disease? This question, far more applicable, if the doctrine were being entertained that this affection resulted from causes native to the soil and climate of Louisiana, presupposes the existence in the city every year of a sufficient number of unacclimated persons for the development, to a greater or less extent, of the disease. But it is immaterial whether the supposition (which is not well founded) be true or not, for observation has shown in the towns that, though the epidemic is invariably preceded by importation of its cause, yet because, most probably, of the absence of a condition or of certain circumstances necessary to the development of this disease, an epidemic does not always follow its introduction. I know of two instances in which merchandise that had been exposed to the infection was imported into Opelousas without affecting others than those who were present on opening the goods. I know of fatal cases occurring in Opelousas and in Washington—in the years 1853 and 1867 in the former place, and in 1837, 1839 and 1852 in the latter place—that did not extend to the attendants on the patients. With regard to these last instances of the disease, they may be regarded as transient cases—that is, unaccompanied with baggage; and therefore, in my opinion, they throw no light upon the ætiology of the fever, which is almost universally admitted *not* to be contagious. I cannot believe it is propagated through effluvia, or emanations arising from the body of a patient. In no instance, in town or country, in this region, has the disease been shown to extend to the attendants on the sick. More than forty cases illustrative of the truth of this statement may be specified, occurring chiefly in the country. Since 1853, there has appeared to be a greater tendency to the spread of the disease in the country.

than previously. Whether this is due to increased malignancy of cause, to a greater negligence in communication with infected places, to receiving visitors with trunks and clothing, or bringing to the country goods from infected places—or, since emancipation, to the constant intercommunication of negroes between town and country, whose insane curiosity, or imagined exemption from the affection, will make them nightly assemble in infected places, whence they return home, fit media of infection—or to all these causes more or less combined—may not be of easy solution. Notwithstanding the necessity of the importation of the cause—of an established focus of infection—yet, the fact appearing that this is often insufficient for a general disease, leads to the inevitable inference that the cause depends absolutely upon certain circumstances for its development. The fact that when imported in any quantity in winter, spring and early summer, it speedily dies out, and can affect only those predisposed to its influence, who may be exposed to it when existing in efficient force, shows, as indispensable, summer heat of a certain duration; and still further, observation shows that something else in addition is required, so as to effect such increase in its power as to engender an epidemic—and this fourth element is believed to consist in a peculiarly vitiated condition of the atmosphere. How long after the commencement of summer heat the local conditions are generated necessary to the preservation, even though in an innocuous form, of the imported infection, is a subject far more worthy of investigation on the ground of practical utility, than for the gratification of curiosity, for the time may soon come when this infection, thus preserved, will meet with all the elements necessary to its full development.

Before closing the subject, it should be remarked that, in our country towns, it requires about two weeks for the cause, whatever it may be, to be so increased as to rapidly spread and cause an epidemic. This circumstance has been so often observed, that it may be regarded as a law in the development of the infection. An individual, for example, with baggage or goods from an infected place, arrives in town. He is sick on, or falls sick soon after, his arrival. The danger to his family will depend on the quantity and malignity of the imported infection. But whether the disease extends speedily to the family or not, it will, as a general rule, be at least twelve days before the fever invades the houses in the immediate neighborhood, and thence spread over town.

It has been my purpose in this article to furnish incontrovertible facts, so that the profession may have additional data on which to base conclusions, as to the manner in which the disease spreads through the country. A number of distinguished medical men still deny or question the importability of the cause of this fever, and sustain the proposition that it is native to the towns of Louisiana, and especially to New Orleans. The facts adduced seem to me sufficient to settle the question that this cause is transmissible from place to place. In proportion to the population of the place, the germ of infection would most probably find stronger adjuvants in its maturation and development, and if it were decided that the place itself, without the assistance of a foreign cause, originated the fever, this circumstance, considering the long intervals which frequently occur between epidemics, would seem to me to be an argument in favor of rigorously-enforced sanitary regulations, including a strict quarantine.

With respect to the malarial origin of yellow fever, it seems to me that different writers on the subject, and especially the late Dr. Nott, of Mobile, in his able support of its animalcular origin, have demolished every foundation upon which the doctrine could rest. It is for the profession to demonstrate the propriety of quarantine, for municipal authorities to make it worthy of confidence by its strict enforcement.

